# The Gut Microbiome and Sex Hormone-Related Diseases

**DOI:** 10.3389/fmicb.2021.711137

**Published:** 2021-09-28

**Authors:** Song He, Hao Li, Zehui Yu, Faming Zhang, Sicheng Liang, Hang Liu, Hongwei Chen, MuHan Lü

**Affiliations:** ^1^The Affiliated Hospital of Southwest Medical University, Luzhou, China; ^2^The Second Affiliated Hospital of Nanjing Medical University, Nanjing, China

**Keywords:** gut microbiome, sex hormones, sex hormone-related diseases, pathogenesis, gut microbial treatment

## Abstract

The role of the gut microbiome has been a hot topic in recent years. One aim of this review is to shed light on the crosstalk between sex hormones and the gut microbiome. Researchers have observed a sex bias of the composition of the gut microbiome in mice and have proved that sex differences influence the composition of the gut microbiome, although the influence is usually obscured by genetic variations. *Via* cell studies, animal studies and some observational studies in humans, researchers have confirmed that the gut microbiome can be shaped by the hormonal environment. On other hand, some theories suggest that the gut microbiota regulates the levels of sex hormones *via* interactions among its metabolites, the immune system, chronic inflammation and some nerve-endocrine axes, such as the gut-brain axis. In addition, bidirectional interactions between the microbiome and the hormonal system have also been observed, and the mechanisms of these interactions are being explored. We further describe the role of the gut microbiome in sex hormone-related diseases, such as ovarian cancer, postmenopausal osteoporosis (PMOP), polycystic ovary syndrome and type 1 diabetes. Among these diseases, PMOP is described in detail. Finally, we discuss the treatments of these diseases and the application prospects of microbial intervention.

## Introduction

The gut microbiome system is the largest ecosystem in the human body. In addition to the large number of microbes in the gut microbiota, gene expression in the gut microbiota is 100 times greater than human gene expression, with more than 1,000 bacterial species in the human colon and each individual host having at least 160 species (Qin et al., 2010; Sommer and Backhed, 2013; Fukuda and Ohno, 2014). The type and quantity of the gut microbiome are closely related to human health. Some scholars even regard the normal intestinal microbiome as a ‘separate organ’ (Possemiers et al., 2011). It is now a consensus opinion that the gut microbiota and host are interdependent. The gut microbiota is necessary for the health of its host and affects the endocrine system, digestive function, intestinal permeability, resistance to foreign pathogens and stimulation of immunisation (Blumberg and Powrie, 2012).

The balance of the intestinal microecological system is affected by a series of factors. External factors include food, drugs and pathogens, and internal factors include immune and endocrine factors. It has been suggested that an imbalance in the gut microbiota can lead to a series of related diseases, especially autoimmune diseases (Blumberg and Powrie, 2012; Tremaroli and Backhed, 2012). Interestingly, recent reports have revealed that many microbiome-related diseases have a sex bias, some of which have been shown to be associated with sex hormones.

Therefore, gaining a better understanding of the interrelationship between sex hormone-related diseases and the gut microbiota is critical to not only help us understand disease pathogenesis but also to target the microbiome and sex hormones for therapeutic purposes.

In this review, we discuss animal and human studies on some gut microbiota-mediated diseases related to sex hormones and the possible mechanisms involved and present evidence for microbial intervention as a treatment for sex hormone-driven diseases.

## The Interaction Between Sex Hormones and the Gut Microbiome

### Sex Bias of the Composition of the Gut Microbiome

It has been widely accepted that the gut microbiome is diverse. Existing studies have noted that this diversity is a result of a combination of factors, including sex differences (Human Microbiome Project Consortium, 2012). In a study including NOD mice (non-obese diabetic), Yurkovetskiy et al. observed that the abundances of Porphyromonadaceae, Veillonellaceae, Kineosporiaceae, Peptococcaceae, Enterobacteriaceae, Lactobacillaceae, Cytophagaceae, Peptostreptococcaceae and Bacteroidaceae were higher in male mice than in female mice (Yurkovetskiy et al., 2013).

To exclude environmental and genetic influences as much as possible and to determine how the sex difference impacts the composition of the gut microbiome alone, Elin Org et al. housed mice of 89 different strains and sexes in separate cages and then sequenced the 16S rRNA of faeces from mice of different strains (Org et al., 2016).

When considering each microbial phylum within the 89 matched strains of mice, Elin Org et al. observed clear differences in the microbiota composition and diversity between sexes, especially in the C57BL/6J and C3H/HeJ strains of mice. In mice from both abovementioned strains, the abundances of Allobaculum, Erwinia and Anaeroplasma were higher in male mice than in female mice. However, when Elin Org et al. examined the entire population together using Bray-Curtis dissimilarity, there were no clear patterns differentiating samples between males and females (Org et al., 2016). These results indicate that sex differences influence the composition of the gut microbiome, although the influence is usually obscured by genetic variations.

### Sex Hormone Levels Regulate the Diversity of the Gut Microbiome

Researchers have speculated that differences in sex hormones may contribute to the sex bias (diversity) of the gut microbial composition, and many studies have provided evidence supporting this speculation (Neuman et al., 2015).

In a cell-level study performed in the 1980s, researchers observed that progesterone could promote the growth of oral *Bacteroides* species and *Prevotella intermedius* (Kornman and Loesche, 1982). Recently, Yurkovetskiy et al. sequenced bacterial DNA extracted from the caecal contents of prepubescent mice (4weeks old) and postpubescent mice (10–13weeks old). They found that α-diversity was not significantly different between the sexes in prepubescent mice. In contrast, they observed an apparent gender bias among postpubescent mice (Yurkovetskiy et al., 2013). Then, they sequenced 16S rRNA genes from the microbiota of male, female and castrated male littermates and observed that the microbiome of females was closer to that of castrated males than to that of males.

Elin Org et al. further proved the effect of androgen on changes using gonadectomy and hormone supplementation (Org et al., 2016). They observed apparent differences in the gut microbial composition between male mice that had undergone gonadectomy and sham male mice, and hormone supplementation *via* a pellet containing 5α-dihydrotestosterone released over 90days eliminated the difference. Furthermore, recent studies have shown that androgen deficiency caused by castration not only alters the gut microbiome but also increased risk factors for cardiovascular diseases induced by hypogonadism and chronic metabolic diseases, such as obesity and loss of thigh muscle mass in high-fat diet-fed male mice. These changes can be reduced when apply antibiotics. These results shed a light on the effect of the antibiotic treatment for curing hypogonadism-induced cardiovascular diseases (Harada, 2018).

Some human studies have also indicated that fluctuations in oestrogen can affect the composition of the gut microbiome, although relatively few related human studies have been performed. Cross-sectional studies account for the majority of studies, whose results are inevitably affected by interference factors, including genetics and the environment, and most of the results can only prove the existence of a correlation between sex hormones and the microbiome instead of a causal relationship.

A few relatively convincing studies have been performed indicating the causal relationship between sex hormones and the microbiome. Koren et al. sequenced stool samples from 91 women and observed that the gut microbiome was profoundly altered during pregnancy, especially during T3 (the third trimester of pregnancy), when oestrogens are at their maximal peak, regardless of health status (Koren et al., 2012). A study from Europe showed that healthy males had a higher abundance of Bacteroides-Prevotella than females, while the microbiota of postmenopausal women did not differ from that of males (Mueller et al., 2006). The results of both studies indicated that oestrogens and related female hormones play an important role in regulating the composition of the gut microbiome.

### The Composition of Gut Microbiota Influences Hormone Levels

It appears that not only is the gut microbiome influenced by sex hormones but also the gut microbiota itself also influences hormone levels.

#### The Gut Microbiome Influences the Levels of Oestrogen, Progesterone and Corticosterone and a Possible Mechanism of These Effects

As early as the last century, researchers observed a decline in oestrogen levels while discovering that antibiotics can affect the composition of the gut microbiome (Adlercreutz et al., 1984).

Recently, Itsuka Kamimura et al. detected and analysed the concentrations of faecal sex hormones in germ-free mice (GM mice), specific pathogen-free mice (SPF mice) and GF-SPF mice (germ-free mice orally administered the faecal microbiome of specific pathogen-free mice). They found that the levels of faecal oestradiol, progesterone and corticosterone in GF-SPF mice were slightly lower than those in SPF mice and that those in GF mice were the lowest (Kamimura et al., 2019). Their results proved that the colonisation of the microbiota in mice can influence the level of sex hormones. One small limitation of their study was that they only focused on mice under 8weeks old and did not measure the hormone levels in postpubescent mice at 10–13weeks of age, similar to Yurkovetskiy et al. (2013).

In human studies, researchers have also found some evidence of the correlation of the microbiome with hormone production. In Flore’s study, the focus was on men and postmenopausal women. By analysing the determinants of oestrogens that are primarily from non-ovarian sources, they found that the levels of total urinary oestrogens were strongly associated with the faecal microbiome richness and α-diversity. These non-ovarian systemic oestrogens were also strongly related with faecal Clostridia taxa, including non-Clostridiales organisms and three genera in the Ruminococcaceae family (Flores et al., 2012).

Associated with previous studies in which researchers found that most gut microbiomes showed β-glucuronidase enzyme activity (Beaud et al., 2005; Dabek et al., 2008), Flores further analysed the relationship between the oestrogen level and the activity of faecal β-glucuronidase. They found that oestrone in urine was significantly related to the functional activity of faecal β-glucuronidase, while other EM (oestrogen metabolites) in urine did not.

By contrast, faecal β-glucuronidase was inversely correlated with faecal total oestrogens. Based on these results, they concluded that β-glucuronidase was an influencing factor of non-ovarian oestrogens (Flores et al., 2012).

Regarding the mechanism, Shen R. L. found that the gut microbiome could produce β-glucuronidase, which could block the binding of oestrogen to glucuronic acid. In this way, β-glucuronidase reduces the inactivation of oestrogen and increases the amount of oestrogen in the body (Shen et al., 2012). In addition, some studies have indicated that enterohepatic circulation is also involved in this process (Holder et al., 2010; Kisiela et al., 2012). In the liver, oestrogens and their metabolites are conjugated through glucuronidation or sulfonation to prepare for excretion in bile (Zhu and Conney, 1998). Conjugated oestrogens are excreted through bile, urine and faeces. Hepatically conjugated oestrogens excreted in bile can be deconjugated by bacterial species with β-glucuronidase activity in the gut (Raftogianis et al., 2000). In this way, β-glucuronidase reduces the inactivation of oestrogen and increases the amount of oestrogen in circulation.

In addition to bacterial β-glucuronidase, some researchers have recently proposed a theory called the gut-brain axis, which may contribute to explaining how the microbiome influences the levels of sex hormones. In this theory, the gut microbiome is seen as a crucial component of the axis, which is involved in the connection between the central nervous system (CNS) and the endocrine system (Cussotto et al., 2018; Osadchiy et al., 2019). Unfortunately, the majority of related studies have been performed using single-sex mice; thus, their focus on the endocrine system is on the HPA axis or insulin secretion, and they do not explain how sex hormones are affected within the gut-brain axis. Therefore, how the microbiome affects the level of sex hormones through the brain-gut axis remains to be explored.

#### The Gut Microbiome Influences the Level of Androgens

There are relatively few experiments that directly prove that the intestinal flora affects androgens. In a recently published article, Hannah et al. detected higher levels of glucuronidated testosterone and dihydrotestosterone but lower free dihydrotestosterone levels in the distal intestine of germ-free mice compared with mice with a normal gut microbiome composition, which proved that gut microbiome plays a vital role in androgen metabolism in mice (Collden et al., 2019).

we still found some potential evidence from research related to type 1 diabetes (T1D). NOD mice are used as an animal model of T1D, and their T1D incidence shows a strong sex bias (female-to-male ratio>2:1; Pozzilli et al., 1993). Castration can increase the incidence of T1D in male mice (Makino et al., 1981), and androgen treatment confers protection to females (Fabbri et al., 2019). These data confirmed the protection of androgens against T1D.

**Figure 1:**
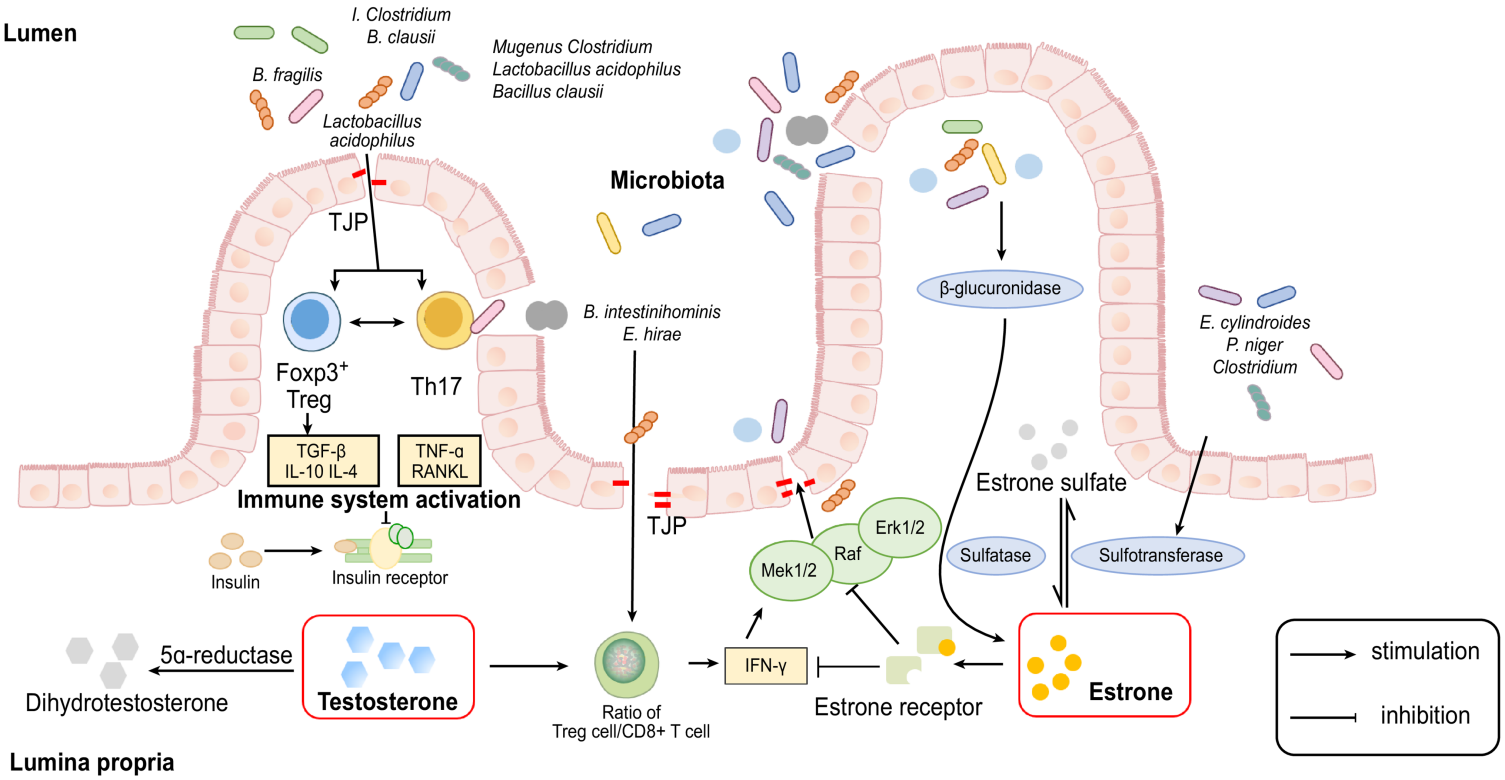
Cellular and molecular mechanisms involved in the interaction between sex hormones and gut microbiome.

Previous studies have shown that germ-free (GM) animals lose the sexual dimorphism of T1D in NOD mice (Yurkovetskiy et al., 2013). However, germ-free mice transplanted gut microbiome from male mice showed increased serum testosterone levels and lower morbidity of T1D (Markle et al., 2013). These results indicate that the protective effect of androgen on T1D is microbiome dependent and highlight the importance of microbial colonisation in modulating host hormone levels, but the specific mechanism remains to be explored (The abovementioned studies suggesting the interaction between intestinal flora and sex hormones were summarised in Table 1; Some cellular and molecular mechanisms involved in the interaction between sex hormones and the gut microbiome were shown in Figure 1).

**Table 1:** Studies on the interaction between gut microbiome and sex hormones.

Model and variable		Hormone changed	Bacteria difference	Results and possible mechanisms	References
Cell study	Resting cell suspensions of pure cultures of plaque organisms from subgingival sample of gingivitis	Progesterone ↑	Oral bacteroides species ↑ Prevotella intermeduis↑	**Result:** Levels of sex hormone regulate the diversity of gut microbiome.	Kornman and Loesche, 1982
Animal study	NOD male mice and NOD female mice	Hormonal differences between two sexes	In male mice: Porphyromonadaceae↑,Veillonellaceae↑, Kineosporiaceae↑, Peptococcaceae↑, Enterobacteriaceae↑,Lactobacillaceae↑, Cytophagaceae↑, Peptostreptococcaceae↑, Bacteroidaceae↑	**Result:** The gender bias of composition of gut microbiome. **Possible mechanism:** Change of sex hormones shape the composition of gut microbiome.	Yurkovetskiy et al., 2013 Org et al., 2016
	Mice from 89 different strains (Male vs female, Examine different strains separately Non-specific hormonal changed)		In male mice: Alobaculum↑ Erwinia↑ aneroplasma↑ (especially in strains of C57BL/6J and C3H/HeJ)		
	Prepubescent mice vs postpubescent mice	Overall hormonal change before and after pubescent	Α-diversity bacteria in postpubescent mice but not in prepubescent mice	**Result:** Levels of sex hormone regulate the diversity of gut microbiome.	Yurkovetskiy et al., 2013 Yurkovetskiy et al., 2013 Org et al., 2016
	Male, female and castrated male mice	overall hormonal change before and after castrated	microbiome of female is closer to castrated male than to male microbiome.		
	Gonadectomy male and Sham male mice before and after androgen supplement	Androgen ↑ or ↓	Androgen supplement eliminate the gut microbiome deviation between gonadectomy and sham mice		
	GF mice, SPF mice, GF-SPF mice	Estradiol,progesterone, corticosterone: the levels of fecal estradiol, progesterone,and corticosterone in GF-SPF mice were a little lower than those in SPF mice and those in GF mice are the lowest.	Germ-free or not	**Result:** The composition of gut microbiota influences hormone levels. **Possible mechanism:** ① Bacterial β-glucuronidase catalyze estrogens convert from conjugated to deconjugated; ② Brian-gut axis.	Yurkovetskiy et al., 2013
	① Sham NOD mice and castrated NOD mice before and after hormone supplement; ② NOD mice, GF NOD mice, female NOD mice before and after microbiota transfer from male NOD mice.	with or without androgen	Germ-free or not; Before and after microbiota transfer	**Results:** ① Castration can increase the incidence of T1D of male mice; and androgen treatment confers protection to females; ② Germ free animals lose the sexual dimorphism of T1D in NOD mice; male-to-female NOD mice showed increased serum testosterone levels, metabolomic changes and increased T1D protection. **The inference obtained from the above experimental results are combined:** The protection of androgen on T1D is microbiome dependent. In other words, microbiome may affect the level of androgen.	Makino et al., 1981; Pozzilli et al., 1993; Org et al., 2016; Fabbri et al., 2019
Human study	Healthy women before and after pregnancy	Overall hormonal change	Gut microbiome got altered during pregnancy especially during the T3	**Result:** Levels of sex hormone regulate the diversity of gut microbiome.	Koren et al., 2012 Mueller et al., 2006
	Healthy men and women before and after menopausal	Overall hormonal change	male had a higher abundance of Bacteroides-Prevotella than female, while post-menopausal women microbiota didn't differed from male one		

*↑, increase; ↓, decrease*.

## The Relationship Between the Gut Microbiome and Some Common Sex Hormone-Related Diseases

### Ovarian Cancer

#### Hypothetical Relationship Between the Pathogenesis of Ovarian Cancer and the Microbiome

The aetiology of ovarian cancer is unknown and may be related to environmental, reproductive, behavioural and genetic factors. Among these factors, dysfunctions of oestrogen levels and the activity of oestrogens are considered important factors. The gut microbiome may be involved in the development of the ovaries by affecting oestrogen levels. Laura et al. found that E2 (17β-oestradiol) treatment could alter the pathophysiology of an ovarian cancer mouse model, leading to earlier onset of tumours, reduced overall survival time and a characteristic papillary histology (Laviolette et al., 2010). Guillermo et al. conducted an *in vitro* study using different ovarian cancer cell lines to confirm the requirement of oestrogen receptors in the aetiology of ovarian cancer. Their results showed that the adhesion and migration abilities of the oestrogen-receptor-positive cell lines were enhanced after oestrogen treatment, while those of the oestrogen-receptor-negative cell line 2,774 EOC were not significantly changed. These results indicated that the effect of oestrogen is directly related to the expression of oestrogen receptors and that oestrogen enhances cell adhesion and migration, which contribute to the metastasis and colonisation of ovarian cancer.

As mentioned above, bacteria with β-glucuronidase activity can mediate the deconjugation of oestrogen to affect the amount of active oestrogen in circulation (see ‘The gut microbiome influences the levels of oestrogen, progesterone and corticosterone and a possible mechanism of these effects’). Combined with the roles that oestrogen level and activity play in the aetiology of ovarian cancer, we can infer that the gut microbiome may contribute to the development of oestrogen-driven diseases, such as ovarian cancer, by affecting the amount of active oestrogen.

#### Clinical Implications of the Microbiome in Treating Ovarian Cancer

Breakthroughs in knowledge of the relationship between the gut microbiome and the chemotherapy efficiency of ovarian cancer have been made using animal experiments. The importance of the gut microbiome in the treatment of ovarian cancer has also been confirmed in many aspects (Chase et al., 2015). Cyclophosphamide is one of the first-line chemotherapy drugs for treating ovarian cancer and can interfere with the synthesis of DNA and RNA. Romain et al. found that the efficacy of cyclophosphamide treatment is related to some species of gut bacteria. The specific mechanisms are still unclear, but they identified that *Enterococcus hirae* translocate from the small intestine to secondary lymphoid organs and increase the intratumoural CD8/Treg ratio, which may participate in the mechanism of action of cyclophosphamide (Daillere et al., 2016).

Viaud et al. found that cyclophosphamide can change the composition of the gut microbiome. In addition, they also observed that cyclophosphamide treatment induced Gram-positive bacterial transfer to secondary lymphoid organs and stimulated secondary lymphoid organs to produce ‘pathogenic’ Th17 cells and memory T cells. In contrast, control group mice with destroyed intestinal microbial homeostasis showed resistance to cyclophosphamide, and the efficacy of the drug was greatly reduced (Viaud et al., 2013).

Based on these findings, we inferred that the content, composition, location and function of the gut microbiome could greatly influence the efficacy of cyclophosphamide. Utilising the role that bacterial β-glucuronidases play in oestrogen metabolism, some researchers regard bacterial β-glucuronidases as possible drug targets for oestrogen-related cancer, similar to ovarian cancer. A conserved motif including asparagine and lysine (N-K) residues has been elucidated in β-glucuronidase, which provides the possibility of using β-glucuronidase inhibitors to increase the chemotherapeutic efficacy and reduce the toxicity of cancer drugs (Wallace et al., 2010, 2015).

We mentioned above that E2, an active form of oestrogen, contributes to the development of ovarian cancer. Regarding the pathway from which E2 is derived, studies have shown that steroid sulfatase (STS) and sulfotransferase (SULT1E1) may play significant roles in the production of E2. STS can activate oestrogens, while SULT1E1 can convert active oestrone (E1) and other forms of oestrogens into an inactive state (Kodama et al., 2011; Purohit et al., 2011). In advanced ovarian cancer patients, researchers have also found a high level of STS and a low level of SULT1E1 (Secky et al., 2013). In addition, there have been reports that the production of STS may be related to some species of the gut microbiome. For example, J Van Eldere succeeded in isolating intestinal steroid-desulfating bacteria from rats and humans (Van Eldere et al., 1988). These results indicate that oestrogen-related cancers can be treated using STS inhibitors or bacterial treatment.

### Postmenopausal Osteoporosis

#### Hypothetical Relationship Between the Pathogenesis of PMOP and the Microbiome

Osteoporosis is a bone metabolic disease taking the form of bone loss and structural destruction. Postmenopausal osteoporosis (PMOP) is a form of osteoporosis that is induced by oestrogen deficiency and leads to an increased frequency of fracture in postmenopausal women. Current studies have suggested the potential and close relationship between gut microbiota and bone remodelling as well as that between gut microbiota and bone metabolic diseases (Collins et al., 2017).

Researchers have found that in germ-free (GF) mice, sex steroid deficiency (sex steroid deficiency was induced by leuprolide) could not induce increased osteoclastogenic cytokine expression, activation of bone resorption and loss of trabecular bone, suggesting that the gut microbiome is crucial in trabecular bone loss caused by sex steroid deficiency. Sex steroid deficiency failed to induce a loss of BV/TV (bone volume density) in GF mice, while it caused a large decrease of BV/TV in Conv. R mice (mice raised under either conventional condition) and Col. GF mice (GF mice recolonized with conventional microbiota), indicating that the process of cortical bone loss caused by sex steroid deprivation is closely related with microbiome (Zhao, 2012).

They further proved that a twice-weekly treatment of sex steroid-deficient mice with the probiotic *Lactobacillus rhamnosus* GG (LGG) or the commercially available probiotic supplement VSL#3 could avoid bone loss. This occurrence may be contributed by the reduction of gut permeability, inhibition of intestinal and BM inflammation. By contrast, supplementation with a non-probiotic strain of *Escherichia coli* or a mutant LGG did not show protection against bone loss.

The above results suggest that gut microbiota disorders may cause increased gut permeability and trigger activation of important inflammatory pathways for inducing bone loss in sex steroid-deficient mice (Li et al., 2016). These results link osteoporosis caused by oestrogen deficiency with gut microbial diversity, intestinal permeability and inflammation.

Recent experiments have shown that many species of the gut microbiome, such as members of the genus *Clostridium* and *Lactobacillus acidophilus* and *Bacillus clausii*, can modulate the Treg-Th17 cell balance, inhibit bone loss and increase bone heterogeneity in osteoporotic mice because Th17 cells secrete RANKL and TNF-α, which are key cytokines for osteoclast formation and are involved in bone resorption (Atarashi et al., 2011; Dar et al., 2018). Treg cells can regulate osteoclastogenesis by secreting TGF-β, IL-10 and IL-4 cytokines too (Talaat et al., 2015; Bozec and Zaiss, 2017).

Oestrogen deficiency may lead to the reduction of some species of bacteria that affect the expression of immune inflammatory cells, such as Tregs and Th17 cells, resulting in increasing bone loss.

By the way, Ang et al. recently found that high-fat ketogenic diets (KDs) could also reduce the levels of gut proinflammatory Th17 cells *via* a gut microbiome-related pathway (Ang et al., 2020). However, whether the KDs will affect the development of PMOP through this Th17-related pathway is still unknown.

Furthermore, some studies suggest that altered intestinal permeability caused by changes in the gut microbiome may be a significant factor for the abovementioned inflammatory response. When high levels of oestrogen interact with the oestrogen receptor (ER) on the intestinal epithelium, the guanosine triphosphate-binding protein Ras and a series of cytoplasmic kinases (Raf, MEK1/2 and Erk1/2) are activated, phosphorylation of some cytoplasmic proteins and transcription factors are induced and the expression of tight junction transmembrane protein (TJP) is upregulated, thereby enhancing the function of the intestinal epithelial barrier and reducing intestinal permeability (Gonzalez-Mariscal et al., 2008).

On other hand, some researchers have proved that oestrogen depletion increases gut permeability, causing some metabolites of the gut microbiome to enter subepithelial tissues as antigens to trigger an immune response, leading to the upregulation of osteoclastogenic cytokines, such as TNF-α, RANKL and IFN-γ (Ye et al., 2003). TNF-α and IFN-γ can reduce the expression of the TJ protein Occludin and increase the synthesis of the TJ cation channel constituent protein Claudin-2 through the Ras-Raf-MEK1/2-Erk1/2 and MLKs- MKK3/6-p38 pathways of the MAPK pathway, leading to a further increase in permeability of the paracellular pathway (Matsumura and Ellington, 2001).

#### Clinical Implications of the Microbiome in Treating PMOP

Recent studies have shown that probiotic treatment can decrease gut permeability by affecting TJ protein expression and enhance the function of the intestinal epithelial barrier, which can reduce the invasion of intestinal pathogenic bacteria and harmful products into the host and reduce the immune response caused by inflammation (Mennigen et al., 2009; Anderson et al., 2010). These results suggest the potential of probiotics for treating PMOP.

Although existing studies have shown that probiotics have many regulatory functions, whether the effects of different probiotics on the host are individual or species-specific remains to be further studied. At the same time, the biosafety, safe and optimal doses and dosage form of probiotics in the treatment of PMOP also urgently need to be verified by corresponding animal and clinical experiments.

### Polycystic Ovary Syndrome

#### Hypothetical Relationship Between the Pathogenesis of PCOS and the Microbiome

Polycystic ovary syndrome (PCOS) is a common endocrine disorder in women of reproductive age whose aetiology may be related to hyperandrogenism, insulin resistance and neuroendocrine dysfunction. There are some studies focusing on the gut microbiome of patients with PCOS, and these studies have found some association between gut microbiome and PCOS. However, the exact mechanisms of the microbes associated with PCOS have not yet been identified.

Some researchers have found that patients who suffer from PCOS have a significantly lower diversity of gut microbiome than that of healthy controls (Lindheim et al., 2017). In the gut microbiome of individuals with PCOS*, Bacteroides vulgatus* is significantly elevated, and the levels of glycodeoxycholic acid and tauroursodeoxycholic acid are reduced (Qi et al., 2019).

By comparing the faecal microbiome, permeability of the intestinal epithelium and inflammatory status of women with PCOS, some studies have proposed a hypothesis that toxaemia resulting from a high permeability of the intestinal epithelium is related to inflammation, insulin resistance and hyperandrogenaemia in PCOS. More in-depth studies are needed to clarify the specific mechanism of endotoxaemia (Lindheim et al., 2017).

Others have suggested that disturbances of the gut microbiome caused by a poor diet lead to a higher gut permeability and more delivery of lipopolysaccharide (LPS) from Gram-negative colonic bacteria into systemic circulation subsequently (Tremellen and Pearce, 2012). The subsequent activation of the immune system may cause interference with insulin receptor function, increase serum insulin levels and then stimulate the ovaries to produce higher androgens and affect normal follicular development.

#### Clinical Implications of the Microbiome in Treating PCOS

Xue J revealed the alleviating effect of inulin and metformin on PCOS is related to anti-inflammation and modulation of the gut microbiota, which may contribute to potential clinical therapy for PCOS (Xue et al., 2019). Diane-35 (oestrogen and progesterone) and probiotics could help to rebuild the diversity of the gut microbiota, and reduction of intestinal flora disorders improved the reproductive function in PCOS-like rats (Zhang et al., 2019a). Moreover, further studies are needed to find out whether manipulation of the gut microbiome can be used as an effective treatment for PCOS.

Transplanting gut microbiota from females with PCOS to recipient mice colonised by *B. vulgatus* resulted in increased destruction of ovarian functions and resistance against insulin, bile acid metabolism alternation and decreased secretion of interleukin-22. As for the possible mechanisms, glycodeoxycholic acid induced IL-22 secretion in three groups of innate lymphoid cells through GATA-binding protein 3. Subsequently, IL-22 improved the PCOS phenotype. These results suggest that manipulation of the gut microbiome, altering bile acid metabolism and increasing IL-22 levels might be a feasible treatment for treating PCOS (Qi et al., 2019). Zhang J monitored observed that the levels of sex hormones and intestinal short-chain fatty acids (SCFAs) increased significantly under the impact of probiotic *Bifidobacterium lactis* V9. Based on these results, they proposed a potential mechanism that *B. lactis* V9 regulates the levels of sex hormones by adjusting the intestinal microbiome in PCOS patients (Zhang et al., 2019b). The above studies provided new insights into the pathogenesis of PCOS, and the potential for using probiotics in PCOS therapy has gradually been noted.

### Type 1 Diabetes

#### Hypothetical Relationship Between T1D and the Microbiome

Abnormal/aberrant microbiota community structures have been reported in many autoimmune diseases (AIDs), including T1D, IBD, asthma and RA (Gianchecchi and Fierabracci, 2017).

T1D is a disorder caused by the autoimmune destruction of insulin-producing pancreatic β cells (Bluestone et al., 2010). The most common symptoms of the disease include hyperglycaemia, polydipsia, polyphagia and polyuria. Currently, patients with T1D require immediate insulin replacement therapy, and the therapy lasts for their entire lifetime. The aetiology and classification of this disease remain unclear and controversial. Current studies mainly focus on the immune system and genetic aspects. The role of the gut microbiome in the pathogenesis of T1D is discussed below.

Part 1, androgen-related:

First, as mentioned above, some studies have found that the gut microbiome is crucial in protecting androgens against T1D, and the protection of androgens is microbiome dependent (see ‘The gut microbiome influences the level of androgens’).

Part 2, the immune response and the metabolites produced by commensal bacteria (short-chain fat acid, SCFA):

In addition, T1D is an AID, and CD4+ and CD8+ T cells, especially Th1 and Th17 cells within CD4+ T cells, are regarded as the crucial point of β-cell loss (Noack and Miossec, 2014). Many studies have suggested that the development of diabetes is related to immune cells, such as Th17 and Treg cells.

L. Ivanov found that antibiotic treatment reduced the differentiation of Th17 cells and increased the number of Treg cells in the small intestine lamina propria in C57BL/6 mice (Uzel et al., 2013). Furthermore, segmented filamentous bacteria (SFB) can induce the differentiation of Th17 cells (Gaboriau-Routhiau et al., 2009). Honkanen et al. (2010) found increase of Th17 cells in peripheral blood T cells from children with T1D, accompanied by increased levels of IL-17, IL-22, retinoic acid-related orphan receptor C isoform 2 and FOXP3 mRNA, which indicated activation of the IL-17 pathway. Their results emphasised the role of IL-17 immune pathway in the pathogenesis of T1D and identified a potential treatment for T1D.

Some studies have shown that there is an interaction between Th17 and Treg cells, and some studies have shown that this interaction may be related to the signal transducer and activator of transcription (STAT) protein family, which includes intracellular transcription factors of cellular immunity, proliferation, apoptosis and differentiation (Fabbri et al., 2019). Previous studies analysing Treg cells in the peripheral blood of T1D patients have brought about some controversial results. Recently, the results of several reports suggest that the frequency of CD4+ FOXP3+ Treg cells in the peripheral blood of T1D patients has not changed significantly (Hamari et al., 2016; Okubo et al., 2016). By contrast, some studies have reported increases and decreases in frequency (Kukreja et al., 2002; Marwaha et al., 2010; Viisanen et al., 2019). Analysis of distinct subsets of total Treg cells may be a future research direction.

Some metabolites produced by commensal bacteria are thought to play important mediating roles in activating the immune response (Arpaia et al., 2013; Rooks and Garrett, 2016). Some researchers have observed that SCFAs may be involved in the pathogenesis of T1D. For example, Sun found that NOD mice had reduced SCFAs, especially butyric acid, compared to those in healthy controls. Similarly, in patients with T1D, there were fewer bacteria producing butyrate than in healthy people (Yuille et al., 2018). These results indicated a close relationship between SCFAs and T1D.

Researchers have long noted that SCFAs can be seen as ligands to bind to G protein-coupled receptors and free fatty acids (FFAs), thereby triggering a series of downstream reactions (Shi et al., 2014). SCFAs can control the production of a cathelicidin called cathelicidin-related antimicrobial peptide (CRAMP), which is secreted by pancreatic islets and has a positive regulation of pancreatic macrophages and other immune cells, maintaining immune homeostasis in the pancreas by induction of Treg cells.

CRAMP is protective against T1D in adult NOD mice, and the protective effect did not appear in female NOD mice. Furthermore, after CRAMP treatment of prediabetic NOD mice, Sun observed that the frequency and number of Foxp3+ Treg cells in the pancreatic islets of prediabetic NOD mice increased, and the incidence of autoimmune diabetes decreased (Sun et al., 2015).

A recent study by Serena showed that butyric acid improved the body’s insulin response (Sanna et al., 2019). The protection of butyrate against T1D may be associated with butyric acid inducing mucoprotein synthesis, which in turn, maintains the tight junction between intestinal epithelial cells and further maintains immune homeostasis (Suzuki, 2013). It has also been shown that butyrate inhibits histone deacetylase (HDAC) activity, reduces the expression of pre-inflammatory factors in dendritic cells and promotes Treg cell differentiation outside the thymus, which affects the development of T1D (Yuille et al., 2018).

However, the protection butyrate against T1D remains controversial, and a more recent randomised controlled trial suggested that oral butyrate did not affect innate immunity and autoimmunity of islet in patients with long-term T1D.

#### Clinical Implications of the Microbiome in Treating T1D

**Figure 2:**
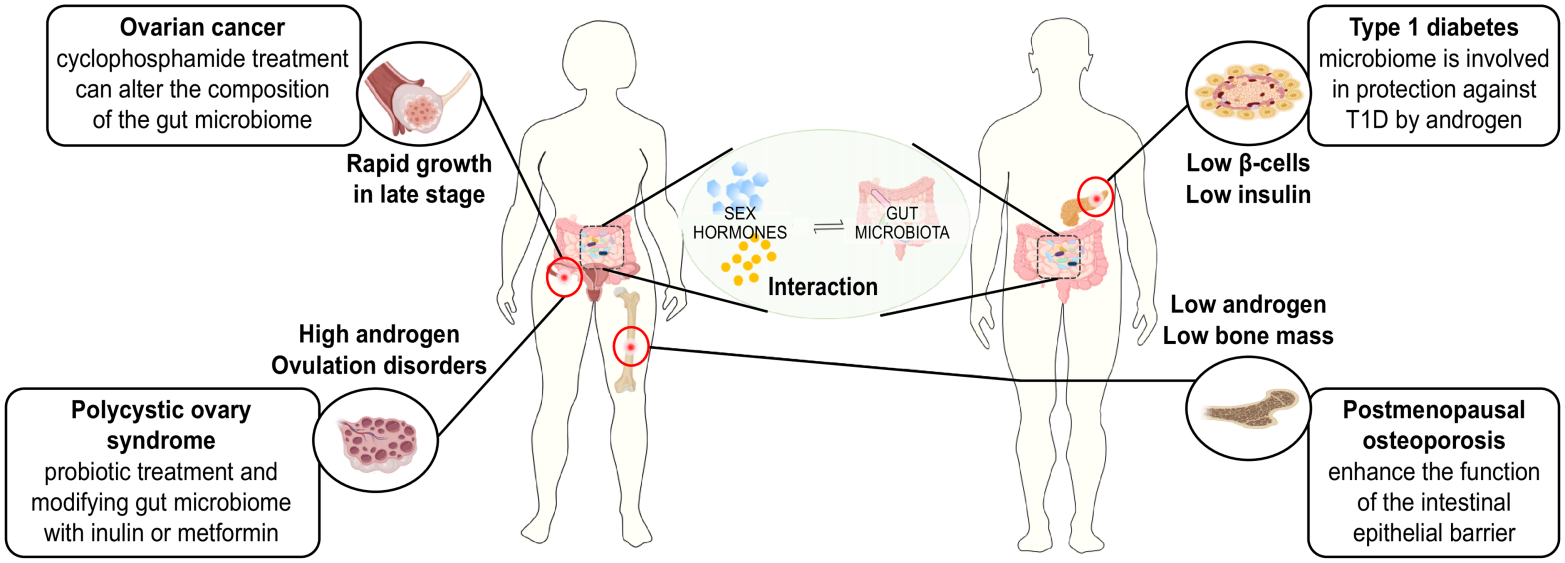
Treating strategies for sex hormone-related diseases based on the interaction between sex hormones and gut microbiome.

For a long time, insulin has been the only choice for treating most T1D patients, but the perspective of the ‘gut microbiome-metabolites-immune axis’ provides inspiration for T1D prevention and treatment strategies. The effectiveness and mechanism of the combined application of probiotics, SCFAs and oral anti-sugar drugs in T1D therapy may be future research directions

(A neat epitome of possible treating strategies for sex hormone-related diseases depending on the interaction between sex hormones and the gut microbiome was shown in Figure 2).

## Discussion

There is a consensus opinion that the gut microbiome plays an important role in maintaining the ecological balance of the human body and mediating the occurrence of certain diseases.

Researchers have observed a sex bias in the gut microbiome and have confirmed the interaction between the gut microbiome and sex hormones, such as oestrogen, testosterone, progesterone and corticosteroids. The most important factor was the relationship between oestrogen levels. Researchers have found possible mechanisms to explain the interaction. For example, it has been shown that gut microbiome-related β-glucuronidases play an important role in mediating the effects of the microbiome on the level of oestrogens, and the brain-gut axis may also be involved in the interactive mechanisms.

We investigated the pathogenesis of several common sex hormone-related diseases and the role of the gut microbiome. Researchers have found that the gut microbiome and its metabolites are involved in the development of these diseases and affect the levels of sex hormones. In addition, the effects of gut microbiome and its metabolites, such as SCFAs, on inflammatory immunity also play crucial roles in the pathogenesis of these sex hormone-related diseases.

The role of the gut microbiome in sex hormone-related diseases is increasingly being studied. However, the mechanisms of synergy or pathways involved in the interaction among the gut microbiome, its metabolites, the immune response and sex hormone levels are still worthy of further study. With the development of technology and the advancement of research, a broader outlook must be taken using microbial supplementation and targeted intervention aimed at investigating the role of the sex hormone metabolism pathway related to the microbiome and its metabolites in the treatment of sex hormone-related diseases.

## Author Contributions

SH contributed to the consulting literature, data collection and drafting of the manuscript. HL, ZY, FZ, SL, and HL participated in the production of pictures, data analysis and made suggestions for revision. ML made suggestions for revision. All authors contributed to the article and approved the submitted version.

## Funding

This work was supported by National Natural Science Foundation of China, Award id 81972296, https://doi.org/10.13039/501100001809 and Southwest Medical University, Award id 00031718, https://doi.org/10.13039/501100014895. Talent Development Project of The Affiliated Hospital of Southwest Medical University. The grant numbers are 20079 and 20061, respectively.

## Conflict of Interest

The authors declare that the research was conducted in the absence of any commercial or financial relationships that could be construed as a potential conflict of interest.

## Publisher’s Note

All claims expressed in this article are solely those of the authors and do not necessarily represent those of their affiliated organizations, or those of the publisher, the editors and the reviewers. Any product that may be evaluated in this article, or claim that may be made by its manufacturer, is not guaranteed or endorsed by the publisher.
